# A Systematic Literature Review of the Teaching Considerations and Practices Provided to Children in an Education Setting with Comorbid Disability and Developmental Trauma

**DOI:** 10.3390/children10081289

**Published:** 2023-07-26

**Authors:** Simone Collier, India Bryce

**Affiliations:** School of Education, University of Southern Queensland, Toowoomba, QLD 4300, Australia; india.bryce@usq.edu.au

**Keywords:** childhood, children, disability, trauma, traumatic injury, maltreatment

## Abstract

Developmental trauma and disability are frequently co-occurring lived experiences for children and young people. The present research explores the considerations and practices for pedagogy provided to children with a disability and who have experienced developmental trauma in an educational setting. A systematic literature review was conducted to explore and synthesise the current evidence base that exists relating to the considerations for quality teaching practice for this cohort of students within schools. Findings revealed five key themes, which contribute to an understanding of considerations and practices for teaching students with childhood trauma experiences whilst considering an additional dimension intersecting with disabilities including mental ill-health. The findings of this study broaden the understanding of the complexities facing the education sector in ensuring inclusion principles are enacted to increase impact and improve outcomes for these students with multiple and complex needs.

## 1. Introduction

A condition that causes disablement can be interpreted as ‘any loss or abnormality of psychological, physiological or anatomical structure or function’ [1]. A disability can be defined as ‘any restriction or lack of ability, resulting from a condition that limits capacity to perform an activity in the manner or within the range considered normal for a human being’ [1]. The term disability is often used to refer to a person’s condition, e.g., a person with a disability. The term used throughout this study refers to a person who experiences disability; however, it is recognised that disability does not result solely from a person’s impairment but from a mismatch between a person’s impairment and the expectations of their environment [2]. Globally, up to 14% of the burden of disease and disability is attributable to mental illness [3].

Children with experiences of developmental trauma are likely to experience poor outcomes across the lifespan [4]. Equally, the risk of exposure to maltreatment is significantly increased when the child has a condition that impacts on their daily functioning. Therefore, the impacts of trauma on the physical, social, emotional, cognitive, and mental developmental domains of a child are equally considered to be disabilities. Thus, developmental trauma and disability are frequently co-occurring lived experiences for children and young people. The present research explores the impact of teaching practices provided to children with a disability and who have experienced developmental trauma, in an educational setting.

### 1.1. Developmental Trauma

Developmental trauma is defined as trauma experiences that occur during in utero, infancy, or early childhood years, and the impact of these trauma experiences undermines normal developmental processes for the child [5]. The trauma may include abuse and neglect and may affect attachment with caregivers, cognitive functioning, self-concept, social relationships, and emotional regulation capacity [6]. Children with developmental trauma experiences face challenges that hinder their academic success, school engagement, relationships, and social and emotional development.

### 1.2. Comorbidity of Maltreatment and Disability

A significant body of empirical research over the last two decades has shown that children with disabilities are at an increased risk for abuse and neglect compared to nondisabled peers [7,8,9]. Children experiencing disability, such as autistic spectrum disorder (ASD), who require mental health intervention or who are receiving support, often report elevated rates of maltreatment, are more likely to be receiving a service from a child protection agency and are at an elevated risk of entering foster care [10,11,12,13]. Comprehending the type, severity, frequency, and duration of trauma experiences for a child in the context of disability is complex given that the notion of causality is challenging to determine [14]. Brown et al. [15] argue that in the context of childhood abuse and neglect experiences, “disability should be perceived as both a predisposing risk factor to abuse and a consequence of it” (p. 113).

The risk of victimisation is increased when a child has a disabling condition or a loss of function due to the complex interplay between biological, psychological, and social factors. To view the maltreatment of children who experience disability through the bio-psycho-social model of disability, all factors, along with the interactions between these elements, are worthy of analysis [16,17]. Considering the abuse of children who experience disability within the context of a bio-psycho-social model of disability and applying this information to Bronfenbrenner’s [18] ecological systems theory of development, a child’s level of risk and/or vulnerability for abuse and neglect is able to be ascertained. Interactions between risk and protective factors in each of the child’s ecologies and the child’s developmental stage and history [19,20] aid in the determination of likely abuse and neglect in the future should no elements in the child’s environments change. Consideration should be given to the child’s characteristics, such as vulnerabilities associated with a disability, along with risk and protective factors that may vary in the proximity to the child (such as proximal being a direct and immediate impact and distal being represented by more social influences) [21]. Risk and protective factors can exist in all ecologies of a child’s life, and there are transactional influences proposed by Ciccetti and Rizley [22], whereby transactions between a child’s characteristics, including their vulnerabilities, and the environment interact. The authors discuss the way potentiating factors increase the risk, whereas compensatory factors decrease the risk [22]. This is further supported by Bolan [20], who proposes a model that states that a child’s likelihood of experiencing abuse is dynamic at any time and that the risk level is determined by the interaction between elements of risk in the macro and exosystems and the child’s developmental stage and history. The macro and exosystems are largely concerned with a family’s beliefs, views, biases, and practices and the community in which they reside. Risk factors that are transactional in nature may include a child’s disability, cognitive functioning, behavioural features, and medical needs, and the environmental factors include parental factors such as drug misuse, parental mental health, employment status, and involvement in crime and community factors such as crime rates, neighbourhood violence, and poor family supports [19].

### 1.3. Disability, Trauma, and Quality Teaching Practices

Children with the combined vulnerabilities of disabilities and maltreatment require empirically supported practices and services to address their unique needs within the educational context. Discussion throughout the literature embraces a common theme that classroom teachers in schools are well placed to identify changes in a child that may be a result of experiencing maltreatment [23,24]. However, the literature also clearly states that teachers are best placed to respond in a therapeutic manner to children who exhibit behaviours indicative of developmental trauma.

The instructional elements of a teacher’s pedagogical practice are often the most considered factor in determining practice quality, as this is assumed to most benefit a student’s academic and/or social and behavioural development [25]. Graham et al. [26] discuss that a student’s test scores are a poor conceptualisation of teacher practice quality and tend to ignore the complex relationships that exist between academic achievement, individual difference, and classroom composition. According to Morris-Mathews et al., [27], a core weakness includes a lack of focus on clear and structured instruction and differentiation necessary to identify and promote practices that are of quality for students with high-incidence disabilities who are present in the classroom but poorly served. This constitutes a significant gap in classroom service delivery for students with disabilities and complex trauma. A common school of thought that underpins some practice frameworks for quality teaching practice is Danielson’s assumption [27], whereby the belief is that “all students have the expertise necessary to design and direct their own learning” ([26] p. 74), argue that this places some students with disabilities and mental health concerns at a distinct disadvantage when teaching approaches and strategies are employed that require students to plan, organise, and be self-directive with their learning. Due to core difficulties with executive functioning, cognitive processing, and emotional regulation, these students require teacher-directed learning that is task-analysed and deliverable in small sequential and repetitive steps with significant relational investment by the teacher.

Currently, in many countries around the world including Australia, systemic inclusive education is being hotly debated as a distinguishing practice from the exclusion, segregation, and integration of children with disabilities in schooling contexts. This includes children with emotional, social, intellectual, mental, physical, and sensory impairments, disorders, and disabilities [28,29]. The most vulnerable children in the Australian context are those with disabilities, those who reside in statutory care, and those who are of Aboriginal and Torres Strait Islander heritage [30]. In a recent lay press publication, published on 22 April 2022, the statistics from Education Queensland reported the suspension rate for children with disabilities to be three times the rate of their nondisabled peers and six times higher for children in statutory care. Children with disabilities accounted for half of all suspensions [28], and disciplinary absences are a reliable indicator that children’s social and emotional well-being and learning needs are not being met in the classroom context.

## 2. Method

The research question posed for the present study is, what are the considerations and teaching practices implemented for students with comorbid disability and developmental trauma? The overarching goal is to synthesise the existing evidence on considerations that inform service delivery and the teaching practices implemented for students with coexisting disabilities and developmental trauma. Additionally, the study seeks to explore the alignment between the practices implemented and the specific needs of this cohort. The findings of this SLR are intended to inform further research into the intersection of the specific needs of this student population and the service delivery they receive in the classroom.

### 2.1. Investigation Strategy

Using the Preferred Reporting Items for Systematic Reviews and Meta-analyses (PRISMA) guidelines as defined by [31], a methodical investigation of peer-reviewed literature commenced in June 2022 using databases EBSCOHost Megafile Ultimate, PsychArticles, Sage, Taylor and Francis, HeinOnline, Wiley, and Science Direct. With the support of the PICO model, a research question was developed [25]. The investigative process that occurred to explore the databases required greater search terms to be developed using the Boolean searches following limited results being identified in initial searches. The search terms were further developed and combined three key areas of interest: (a) “disability and children”, (b) “trauma or maltreatment”, and (c) “teaching practices”. Following the database searches being completed, duplicates were removed, the remaining studies were screened for relevance to the research question, and the reference lists of the relevant hits were inspected for additional studies. The results of the manual reference list investigations were then screened for applicability, and duplicates were eradicated. Further exploration included citation searches of applicable data from previous searches and analyses of reference lists. Peer-reviewed papers and those published between January 2010 and June 2022 were the criteria for inclusion.

### 2.2. Papers Selected and Quality Assessment

Cohen’s [32] method of preview, question, read and summarise (PQRS) [11] was the framework utilised to select papers for this review. The preview stage was engaged to explicate article titles and abstracts and to sort the studies as qualitative, quantitative, or mixed methods. All three types of studies were considered, as the primary focus of this SLR is to determine the evidence of current quality teaching practices for students with disabilities and developmental trauma. During the enquiry stage, all studies were evaluated against the criteria for inclusion. It was determined that papers that met the benchmark to be included had all three criteria of the focus of the research question (i.e., disability and children, trauma or maltreatment, and teaching practices), were full-text articles, and were studies published in English.

**Figure 1:**
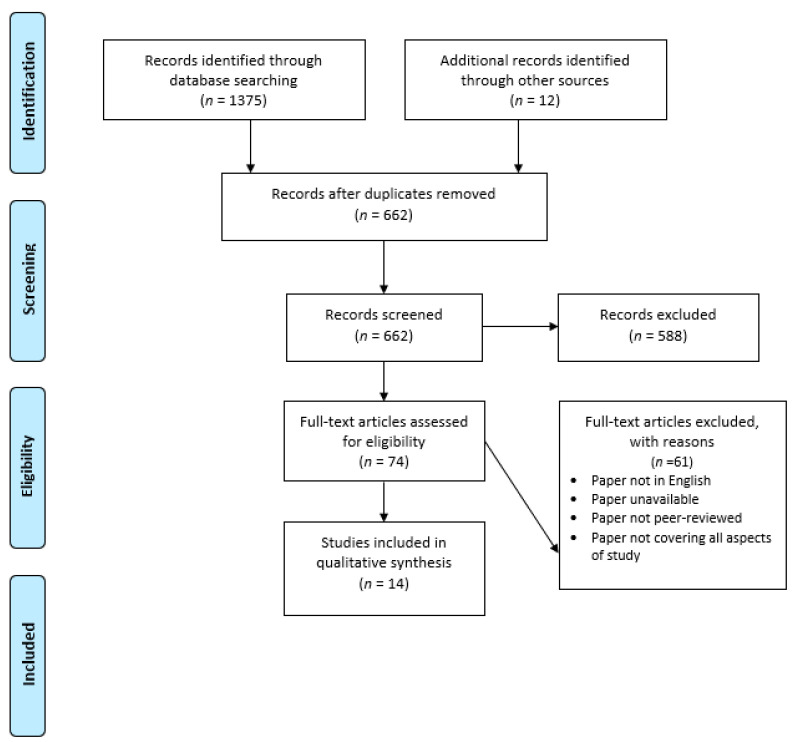
PRISMA Flow Chart.

The investigation and article additions are represented in Figure 1 as a PRISMA flow chart.

Fourteen articles met the criteria for consideration in this study and were compartmentalised according to their research strategy. The qualitative studies (*n* = 12) were analyses and rated against the Critical Appraisal Skills Programme (CASP); see [33]. Qualitative studies were analysed against the following criteria including aims, methodology, design, population samples, collection of data, ethical considerations, analysis, results, and impact. The single mixed-methods study was collated with the qualitative studies based on its predominantly qualitative methodological approach (Table A1, Appendix A).

Quantitative studies (*n* = 2) were analysed and rated using the Strengthening the Reporting of Observational Studies in Epidemiology (STROBE) [34] evaluation instrument to evaluate the significance and worth of the cross-sectional studies. Despite the presence of low-level bias, these quantitative studies were deemed relevant and valuable to the research question. The final number of studies included in this systematic review consisted of 14 studies (Appendix B).

### 2.3. Triangulation

Systematic literature reviews require rigour, transparency, and impartiality along with an uncompromising approach to reduce bias and to ensure replicability [35]. To enhance the credibility of the results, the investigative strategies were replicated as part of the assessment processes by an independent researcher. This resulted in discovering the same findings through their investigation and analysis.

### 2.4. Data Synthesis and Emerging Themes

To support the identification of patterns throughout the fourteen studies, thematic analysis was employed to assist in determining emerging themes from the literature. With the research question in mind, the six phases of thematic analysis proposed by Braun and Clarke [36] were implemented in a non-linear approach. To assist in the analysis and synopsis phase, a synthesis table was constructed (Summary C). This table facilitated the classification of the 14 studies that comprised the data set [37] following the researcher’s familiarisation with the literature and the noting of any initial analytical features. The data sample was then re-reviewed, and initial codes were extracted to capture conceptual patterns in the data set [36,38] from which the researchers constructed the second-order themes. These themes were then reviewed in relation to the coded extracts and the full data set, further defined, and named to identify third-order themes [36,38]. These themes were further reviewed to assess if they reveal a convincing narrative. Further analysis occurred amongst the researchers to determine if second-order themes required splitting or collapsing to commence third-order theme development. Following the establishment of the third-order themes, the essence and nature of each theme were evaluated further and named [36] (Appendix C).

The synopsis of the fourteen studies resulted in five focus themes: mental ill-health caused by trauma contributing to disability; inclusion and addressing the gap caused by integration; quality teaching encompassing social, emotional, and behavioural skills; and cultural responsiveness. The analysis of these themes is depicted in Appendix C.

## 3. Results

This SLR examined the teaching practices that are provided to children in an educational setting who have experienced disability and childhood maltreatment. The key findings identified five core themes: mental health caused by trauma contributing to disability; disability is a risk factor for childhood abuse; capacity of classroom teachers to achieve inclusion; quality teaching encompassing social, emotional, and behavioural skills; and cultural responsiveness.

### 3.1. Mental Ill-Health Caused by Trauma Contributes to Disability

A consistent theme throughout all studies that were analysed as part of this SLR (*n* = 14) was the impact of repeated maltreatment and polyvictimisation on the mental health of children and young people with disabilities. Stewart et al. [39] discussed that for children and young people with disabilities who experience significant maltreatment that was ongoing, a likely outcome is poor mental health, which often further compounds the pre-existing disability. Additionally, a number of studies (*n* = 4) identified that children experiencing disabilities that include mental health concerns are at greater risk of school failure; referral to special education units; receiving disciplinary referrals, suspensions, and expulsions; absenteeism; and disengagement from school [40,41,42,43]. Two studies [14,41] identified that children and young people who are exposed to childhood trauma are at significantly greater risk of mental health instability, academic problems, emotional and behavioural disorders, sexually risky behaviours, and substance misuse, with one in five of these children having a diagnosable mental health condition that will cause severe impairments across the lifespan. Larson et al. [41] described children experiencing disability as being part of a minority group that disproportionately does not often receive treatment or receive a diagnosis. Untreated mental health conditions can lead to severe disabilities or even death from suicide [41]. This is further supported by Bucker et al., [44] who noted that the co-occurrence of childhood trauma with psychiatric disabilities and disorders is associated with higher rates of suicide attempts.

Bucker et al. [44] explored the relationship between exposure to childhood maltreatment and cognitive disabilities. Cognitive disabilities occur when there are impacts on the child’s development from an early age caused by childhood maltreatment and often correlate with significant psychopathological symptoms. This is further supported by Merritt and Klein [45], who discuss that mental health disorders are likely to impact emotional development, behaviour, speech, and the capacity to exercise higher-order cognitive functions including behavioural and emotional regulation, evaluation of risk, and language and communication skills.

### 3.2. Disability as a Risk Factor for Childhood Abuse

There is evidence throughout the literature (*n* = 5) that indicates children who are living with mental health and physical health disabilities are more at risk of abuse than neurotypical children [40,43,46]. Simpson et al. [43] also commented on the likelihood of children with cognitive disabilities as being three to four times more likely to be abused and neglected than their peers without disabilities. Quill and Kahu [42] discussed that below-average cognitive functioning was a common pre-cursor for sexual abuse for children, and children and young people with disabilities that predate the trauma experiences often end up in special education classes and have three times the dropout rate of their peers. According to Simpson et al. [43] children with mental and physical disabilities and trauma backgrounds require greater levels of protection than their nondisabled and non-traumatised peers.

### 3.3. Capacity of Classroom Teachers to Achieve Inclusion

Overwhelmingly, the literature (*n* = 9) posits that classroom teachers feel ill-equipped to manage the diverse needs of children with diagnosed conditions, developmental delay, psychopathology, socio-emotional behavioural conditions, and atypical development including sensory, communicative, physical, adaptive, cognitive, and motor domains. The implications for children who arrive at school with significant developmental delays and mental and physical health disabilities are that teachers and school-based social workers are ill-equipped to meet their specific needs in the mainstream classroom [26]. In the study conducted by Simpson et al. [43] it was found that the intersection that exists between the discernment of disability and the features of abuse and neglect is complex, interferes with assessment outcomes, and requires highly skilled practitioners to have effective communication skills to determine what is impacting on the child. Other imperative qualities of practitioners that were identified included practitioners being able to support students who have experienced trauma and who have a disability, implement child-focused assessments, have the time to work with augmentative communication styles, connect with the child’s network, and engage in tailored support planning [43].

Simpson et al. [43] found that disability status (both mental illness and physical health) is often disregarded in the assessment phase of suspected maltreatment despite the disability status being a risk factor for victimisation. Teachers identified knowledge of, training in, and access to effective assessment tools for young children as a challenge and report a lack of knowledge when supporting children with disabilities and mental health issues in their classrooms [45]. This significantly influenced the authenticity of inclusion practices for children experiencing concurrent disability and trauma.

Adrihan et al. [47] discussed the need for a strong coaching model to be available to school staff to support inclusion at the assessment, intervention, and collaboration phases. This practice is designed to support classroom teachers and administrative staff to be able to identify the needs of the students and engage teachers in a mentoring and coaching model to learn new skills to support this cohort of students to be fully included rather than simply integrated into the fabric of the school. This was further supported by [43] who discussed that professionals often chose to define child abuse from their own understanding, and this can impact adversely on service delivery in the classroom.

Ferguson and Wolkow [40] discuss how children with disabilities and experiences of trauma are marginalised at school and believe children with needs for specialised support have their educational needs neglected or, at best, given minimal attention. According to Simpson et al. [43] when assessment and intervention occur via multi-disciplinary teams and when staff receive regular training and coaching in child maltreatment and trauma-responsive approaches, there is a greater likelihood of inclusion occurring for the students.

### 3.4. Quality Relational Teaching Practice Encompassing Social, Emotional, and Behavioural Skills

Numerous studies (*n* = 8) in this review refer to the social, emotional, and behavioural skills that are required to be explicitly taught, on a repetitive basis, to children and young people experiencing disabilities who have been exposed to early traumatic experiences and subsequently have mental illness and subsyndromal symptoms of psychopathology. It is deemed important for this to occur within a relational practice framework [43]. Teachers need to implicitly understand the attachment disruptions and neurological and biological implications for children experiencing disability, coupled with mental health issues as a consequence of childhood trauma [42]. Teachers with this insight tended to have compassionate trauma-informed responses to the behavioural escalations that result from a nervous system that is struggling to regulate [48]. Further to this is the important understanding teachers gain when they become familiar with the brain’s architecture and the impact that disabilities and trauma can have on the brain’s functioning.

Two studies [26,40] discussed the high degree of quality teaching practice required to cater to the emotional, social, and behavioural needs of students with high-incidence disabilities, such as ADHD, learning disorders, and autism, as the majority of these students are educated in a mainstream classroom where integration is the norm, and an absence of genuine inclusion is apparent [49]. Graham et al. [26] highlighted the relational approach being assumed by a teacher aide to support students to experience inclusion as opposed to integration. A student’s social and emotional needs are more likely to be addressed when a close connection and trusting relationship can exist in the classroom with a key adult [48]. Robinson [48] revealed that students experiencing disabilities who have mental health concerns as a result of maltreatment were found to be more likely to regulate their emotions with the confidence of a secure base with their aide, thus having a positive impact on the behaviours demonstrated in the classroom by the student.

### 3.5. Cultural Responsiveness

Another key theme that was consistent throughout included Indigenous children being over-represented as vulnerable in education, health, and disability sectors and the need for child-focused and culturally responsive intervention [41,43]. This systematic review located four articles (*n* = 4) that discussed the impacts of entrenched racism amongst Indigenous races across the globe. Discussions related to the intergenerational transmission of harm and parental outcomes, in particular, the barriers Indigenous families need to overcome to be able to engage in early education whilst addressing family cultural needs and targeting developmental delays. Farnfield and Onions [50] addressed the patrimonial effects of early life trauma, whereby intergenerational effects of childhood trauma can have significant impacts on parents raising their own children. These effects are generally seen in their attachment styles, risk-taking behaviours, and mental health stability. For the purpose of this review, intergenerational transmission makes reference to the modelling that may be demonstrated through experiences of symptoms, reactions, patterns, and behaviours from historical experiences of interfamilial adversity and also through the way genetics are altered and passed down through the generations. Ref. [46] discussed the current reduced likelihood of Indigenous young people completing secondary school as a result of entrenched racism. Further, they highlight the impact the Australian ‘whole-of-government’ policy, the Closing the Gap Strategy, is intended to have on education, health, and disability. The significant gap of 24% that remains between Indigenous and non-Indigenous students completing school according to [46] is relatively consistent with other nations including New Zealand [43] and the United States [41]. Larson et al. [41] further highlight that 70% of children with mental health disorders do not receive mental health services, with minority groups and lower-socio-economic youth disproportionately represented.

According to Macniven et al. [46], the National Framework for Protecting Australia’s Children 2021-2031 supports commitments made under the Closing the Gap Strategy and the four priority reform areas, which focus on children and families with multiple and complex needs, Aboriginal and Torres Strait Islander children, children and young people with disabilities, and children and young people who experience maltreatment, including those in out-of-home care. This paper targets the student cohort that fits within these four parameters.

## 4. Discussion

The overall purpose of this review is to consider the pedagogical practices implemented with children and young people in an educational setting who experience disability and have endured childhood maltreatment. We now interpret the findings in relation to the initial aims and research question. The review revealed five key themes that highlight the key issues impacting quality practice when teaching this student cohort in the classroom: (1) mental health conditions caused by trauma contribute to disability, (2) disability is a risk factor for childhood abuse, (3) capacity of classroom teachers to achieve inclusion, (4) quality relational teaching practice needs to encompass social, emotional, and behavioural skills, and (5) cultural responsiveness. These themes can be categorised into considerations and quality teaching practices and will be discussed explicitly in the following discussion.

### 4.1. Considerations for Educators

The often-overwhelming realities for educators teaching students with a disability caused by maltreatment or that is additional to a pre-existing disabling condition are far-reaching for the teacher, the student, and for the other classroom members. The notion that mental ill-heath caused by trauma can further contribute to a disability and acknowledging that a child’s disability presents as a risk factor for future maltreatment add to the teacher’s load when considering student welfare, differentiating classroom content, and implementing differentiated discipline strategies. The literature supports a coaching model to assist the competency of educators to enable greater confidence in planning for and implementing programmatic adaptations to ensure inclusion is a priority for each student. Further to this, a quality collaboration between educators and community agency staff who specialise in the mental health and developmental needs of the chronological and developmental stages of students is suggested [49]. A key focus of all practice considerations when working with students with mental illness is to ensure that classroom teaching practices do not inflict further trauma or activate past traumatic experiences [47]. This often unintentionally occurs when teachers comply with behavioural approaches rather than trauma-sensitive responses to challenging behaviours. There is, therefore, a need for school-based participatory action research and a commitment to staff learning about interpersonal trauma, instructional biases, and a whole-of-school approach to a cultural shift towards trauma-informed positive behavioural support [26]. Research has demonstrated that when schools adopt change processes and focus on building staff and student resilience, student drop-out rates decline, and recidivism rates of challenging behaviours reduce as revictimisation is lowered [51,52].

### 4.2. Quality Teaching Practices

This research revealed the key themes that impact on quality teaching practices for students with comorbidity and includes the capacity of classroom teachers to achieve inclusion and quality relational practice and the need to incorporate social, emotional, and behavioural skills whilst being culturally responsive.

The process of inclusion is unquestionably being attempted; however, in its truest form, it is not occurring for these students. Further, it may be argued that it is further marginalising the minority groups who are contending with socio-economic disadvantage [40]. The deleterious impacts of maltreatment on a child’s brain development have significant implications for children and young people with disabilities and their inclusion in a mainstream classroom context. Disability when considered in the context of education needs to include both developmental disabilities and mental illness and disorders impacting children who have experienced childhood abuse and neglect. This comorbidity must be considered as a credible need to be addressed within the parameters of “*special needs*”. The literature revealed that gaps exist in quality teaching practices for students with high-incidence disabilities who exist in a mainstream classroom as part of the inclusion model [25,26,53]. The concept of intersectionality asserts that children with abuse and neglect experiences have mental health concerns, and these do not exist independently of each other. These two markers (mental health and trauma) when intersecting with disabilities result in greater disadvantage, risk, oppression, and complexity. The intersectionality of disability, mental illness, and childhood trauma is essential to understand to ensure they are considered when developing reasonable adjustments for the student in the classroom to accommodate these incommodities.

This systematic literature review identified change processes that focus on new templates for the teaching workforce at a practice level and at a systemic level, which is highlighted in the results of this review. These change processes address staff being more intentional in approaching the practice of teaching, challenging antiquated practices and belief systems, measuring the impact of relational teaching approaches with marginalised student populations, and addressing the notion of inclusion vs. integration that is still arguably occurring in most mainstream classrooms. The literature review unveils the implicit racial bias that is present in schools related to minority groups including the Australian Aboriginal and Torres Strait Islander cohort of students and discusses the results, highlighting explicit approaches to improve practice quality to enhance the impact and outcomes for this vulnerable population. Goodwin [47] suggested teaching practices and considerations that may be suited to this cohort of students as including seeking alternative strategies including video modelling; creation of podcasts; challenging adult thinking beyond the current context, asking “Why is this student struggling to interact with the environment/context right now?”; and bringing a sense of calm to the instructional context, which facilitates relational building and connections where attunement with the student becomes possible. Ensuring that task analysis occurs is an important consideration for achieving quality teaching, as step-by-step instruction whereby the student can accomplish smaller chunks or singular tasks allows for increasing competency for larger tasks [42].

The theme relating to inclusion and addressing the gap caused by the process of integration can be mitigated through the suggestions made in the literature. These include opportunities for increased involvement in school operations, forging a sense of ownership and belongingness to the student’s context, empowerment of students through opportunities provided through sports, citizenship, and community participation; personal skill development; adaptive functioning; capacity building and evidence of an increased sense of belongingness; and connectedness and commitment to their school and community [42]. The theme relating to mental health conditions caused by trauma contributing to disability is addressed through the implementation of these strategies and enhances the impact of quality teaching provided to students with a mental illness caused by maltreatment and who have a disabling condition. Other key suggestions focus on the inclusion of minority groups such as children and young people with disabilities and those from a minority background and the importance of culturally adapted interventions and strategies such as cultural storytelling and high levels of family involvement in the school. This may include a focus on positive psychosocial development including support groups for parents involving particular projects on the school campus, the development of job descriptions for parents to assist and support in the classroom, informing parents of opportunities to gain information to facilitate parental involvement becoming more accessible, and working with parents to match parental availability and interests with the needs of a classroom [54]. Other key suggestions to help enhance the impact of high-quality teaching to students with mental illness is the inclusion of traditional beliefs and stories including child-centred parenting practices supported by the school; culturally adapted play therapies to build self-esteem, identity, and confidence and to explicitly teach positive coping skills; and rewards-based teaching [46].

### 4.3. Implications for Research and Practice

The aforementioned themes can be utilised to inform the implementation of quality teaching practices and considerations for students experiencing disabilities and childhood trauma to ensure they are adequately assessed and programmed for and receive the interventions and specialised classroom practice that meet their complex needs. The result of this study suggests there may be a benefit in more comprehensive screening and assessment procedures in educational contexts for children and young people with comorbid disability and trauma to address the risk of adverse outcomes such as risk-taking behaviours, juvenile delinquency, and mental illness. Within mainstream classrooms, consideration for culturally sensitive and trauma-responsive tailored supports to address complex cumulative harm is suggested. The possibility of revictimisation across the lifespan will further compound the effects of disadvantage. Prosocial coping strategies and proficiency in acquiring functional skills, coupled with disability-, trauma-, and culturally specific wraparound supports earlier in a multi-disciplinary school-based centre, are considerations for policymakers and practitioners. Further research to implement and evaluate specific interventions, assessments, and practices that target this vulnerable population is proposed.

## 5. Limitations

It is acknowledged that this review has a number of limitations including the assumption that the studies reviewed have a valid methodology and that the results are legitimate. The articles used in this review encompass a range of methods, assessment criteria, and subscales. Given the diversity of terminology to describe quality teaching practices, disabilities, and childhood maltreatment, there is a possibility that some terms and phrases have been missed. There is no discussion relating to race, ethnicity, and gender, as these were not elements reported in the papers reviewed, and, therefore, assumptions cannot be made about these factors and their impact on teaching considerations.

## 6. Conclusions

With consideration of the limitations of this study, the findings highlight the considerations and practices that may support vulnerable students who have suffered childhood maltreatment and have disabilities. The five core themes that have emerged from this study highlight the need to consider disability as a contributor to trauma, as well as a risk for future maltreatment, to adequately appreciate the needs of children experiencing comorbid disability and trauma. These considerations emphasise the need for risk assessment and protective behaviour strategies to be implemented to support this specific cohort and to improve lifespan outcomes. The findings of this study revealed that student outcomes are likely to improve when quality teaching practices and systematic change occur and implicit cultural bias is reduced. Further research on teaching practice quality to ensure that inclusion is prioritised for this cohort of students and that integration processes are no longer the norm will enhance outcomes for students experiencing comorbid disability and trauma in mainstream classrooms.

## Data Availability

No newly created data constitutes this study.

## Appendix A

**Table A1:** CASP Qualitative Table.

Reference	Quality of Resource (Peer-Reviewed)	Participants	Aims of Study (Underlying Arguments)	Methodology (Research Design)	Limitations	Results (Themes)	Conclusions of Paper
[40]	Peer-reviewed	Children and youth in care	Critical examination of education issues that result in children in care experiencing school failure and long-term involvement in the justice and social services systems.	Analysis of the qualitative and quantitative literature on school outcomes and barriers to educational progress for children	Use of Canadian data only	Improve interagency collaboration and make reasonable adjustments to address the special needs of the child pertinent to the disability and the impacts from the maltreatment before addressing learning. Social and emotional developmental needs to be the priority. Staff training and development to enhance school stability, placement stability, and belonginess is a priority.	Children who have special education needs as a result of maltreatment, require child-centred approaches as part of their education planning process that is largely relational in nature
[47]	Peer-Reviewed	Children from birth–age 3 with developmental needs	To determine changes to screening and evaluating methods impacting eligibility and the identification of social and emotional concerns	Qualitative analysis of a change in a screening and rescreening process	Access to parents was limited and challenging at times	Greater time commitment from the staff to engage families Infant and child outcomes improved when families receive support throughout infancy Multi-agency collaboration with similar expectations Mental health services for children required	Complex trauma assessment confirmation to become a criterion for automatic eligibility/diagnosed condition to access services. The trajectory for children with complex trauma is well known and requires intervention as the likelihood of developmental delay is significant.
[42]	Peer-reviewed	Seven female Indigenous teacher aides, aged 46–50 years.	Evaluate the effectiveness of teacher aide relationships with children with multiple and complex mental health needs and disabilities in a mainstream classroom setting	Exploratory Study	Inside researcher	Special relationships can exist with a teacher aide that often does not with a classroom teacher. Relational approach is essential as a precondition for learning to occur. Pathway to engaging in learning is belongingness.	Implications for practice include centrality of teacher aide–student relationship to engagement, attendance, regulation, and learning
[48]	Peer-reviewed/ conceptual paper		Connections to violence and drug misuse	Participants self-reported, NYSFS analysed using descriptive statistics in a longitudinal study		Drug misuse is connected to abuse types—physical and emotional	Parental violence, parental mental health, and parental drug misuse impacts on a child’s future vulnerabilities in these areas
[26]	Peer-reviewed	50 Grade 7–10 students with a history of disruptive and disengaged behaviour from 3 secondary schools serving disadvantaged students.	Assess student perceptions of 4 domains of quality teaching	Interviews	Participants only taken from Queensland schools, rather than a representative sample across Australia.	~ Current frameworks of practice do not have the micro detail required for teachers to meet the instructional needs of children with disabilities/trauma in mainstream classrooms ~ Quality instruction does not equal quality for all	Teacher support for learning was deemed more important to students than emotional or welfare support. ~ 3 elements identified as comprising quality teaching including classroom organisation and emotional and instructional support ~ Inclusive quality teaching is about dosage and engaging in instructional practices that are consistent, frequent, and intensive enough to support learning.
[46]	Peer-reviewed	Preschool aged Indigenous children	Determine quality education strategies to support Indigenous preschool-aged children with trauma backgrounds and developmental disabilities	Analysis of international literature on adapted interventions to improve cognitive, emotional, and developmental delay among young children	Three studies, small numbers in each study. Heterogeneity of study design	Four key themes to support preschool-aged children with mental health and developmental disabilities included storytelling, family involvement, culturally adapted CBT, and rewards-based teaching	Teaching strategies and pedagogical approaches to support Indigenous children with mental health instability and disabilities are not occurring in mainstream classrooms
[43]	Peer-reviewed	Focus groups followed by semi-structured interviews	Eight disability-aware teachers (focus groups) and allied health professionals followed by 4 school social workers (interviews)	Exploration of experiences and perspectives of professionals in developmental trauma and intellectual disabilities	Small sample size and challenges associated with transference of findings to other cultural backgrounds that are not Māori or Pacifica.	Two core themes: (a) intersection of disability knowledge and skills; (b) relevance of relational practice	1. Importance of practitioners utilising core practice frameworks, employing multi-disciplinary skills and knowledge, understanding bi-cultural practices and understanding nuances related to cultural disability and trauma. 2. Communication and child-focused and culturally sensitive rigorous assessment tools. 3. Expert knowledge level of disability/trauma and open to engaging in the education to develop skills
[51]	Peer-Reviewed	Therapeutic Residential school setting	Self-regulation assessments of children	Child Attachment and play assessment	Small sample size of participants	Childhood trauma is impact by capacity to self-regulate	Abuse types and capacity to self-regulate in classroom settings
[50]	Peer-reviewed	Cohort of teens (10–17 years) in OOHC, Tasmainia	How do the trajectories of some children in OOHC travel compared to others who are also part of the child protection system	Life story analysis		High levels of cumulative harm equate to psychopathology	Cumulative impacts of harm not considered due to episodic system setup
[55]	Peer-reviewed	Primary and special education teachers and school counsellors	Review article on the intersection between attachment and neurobiological and complex trauma	Trauma counselling field provides essential insights into educational outcomes	Canadian and American contexts only	Lifespan implications for cumulative harm contributing to complex mental health	1. Immature internal neurological and biological systems are linked to ongoing impairment of attachment and neurobiological integrity. 2. Neurological changes occur as a result of developmental trauma, providing insights into effective interventions for the learning context in the future
[14]	Peer-reviewed	8980 child participants (4156 with maltreatment history) aged 4–18 years from 50 mental health facilities in Ontario	Examination of relationship between polyvictimisation and risk of harm to self and others	Semi-structured interviews at intake into mental health facility. Additional information gathered from medical records		Cumulative relationships exist when experiencing multiple types of maltreatment. Cumulative risk exists in relation to the harming of oneself or others	Importance of background assessments when psychological injury occurs that considers all forms of maltreatment to understand risk of harm and to inform intervention and trauma-informed practices that need to occur in the classroom.
[41]	Peer-Reviewed	4 main topics for the review	Extensive review of empirical studies based on a hypothesis generated through a conceptual model	Cross-sectional databases that utilised valid and reliable surveys	United States context only and may have been limited by search terms and urban contexts	Themes: 1. Chronic childhood trauma and educational impacts. 2. Mental health care disparities	~ School-based health centres to address disabilities, mental health, physical health, trauma, and poor academic achievement. ~ Access, quality, utilisation, and funding of school-based services was found to have disparity amongst ethnic minority groups. School-based centre that used a counsellor had students improve overall grades.

## Appendix B

**Table A2:** STROBE Quantitative studies assessment.

Study	Title/Abstract	Introduction	Methods	Results	Participants	Summary and Outcome Measures	Bias/Generalisability	Overall Quality Based on Authors’ Independent Review. Research Is of Value.
[42]	Do early care and education services improve language development for maltreated children? Evidence from a national child welfare sample	High	High (Survey)	Medium—Worker continuity. Large percentage of original sample excluded due to missing data. No information relating to how long children participated in ECE or at what level of intensity (dosage) nor any differentiating between types and/or quality of ECE programs.	High	High—Young children who have developmental trauma and were placed into an ECE had better language development outcomes at 18 months. ECE can improve a child’s (with complex trauma) ability to comprehend and communicate language and buffer children against negative developmental outcomes including impairments and delays	low	High—Higher-quality and targeted ECE programs produce better developmental outcomes for children with developmental trauma and associated disabilities/developmental impairments. Children who have endured supervisory neglect scored higher at the conclusion of the ECE program, placing them in the normal limits for early language functioning
[13]	Cognitive Impairment in school-aged children with early trauma.	High	High—Assessment of broad cognitive domains including learning, working memory, executive functioning, attention, verbal/premorbid intellectual functioning, and impulsivity	Children exposed to early childhood trauma required differentiated curriculum and discipline responses that are differentiated and trauma-informed.	High—30 × children aged 5–13 years with a history of early severe trauma. 30 × age and gender matched controls (children without trauma experiences)	High—Despite many not meeting the criteria for diagnosis of disorders, the subsyndromal symptoms indicate far greater challenges with cognitive tasks than children not exposed to trauma.	medium	School-aged children exposed to early trauma show worse performance on attention, immediate verbal recall, and working memory tests than the control group. Trauma group showed a higher prevalence of subsyndromal symptoms than the controls, and this is associated with worse cognitive functioning. Subsyndromal symptoms are associated with impairment in attention and in estimated IQ—effects of trauma on cognition may in part be explained by subsyndromal conditions. Attention is more chronically impaired than other cognitive functions in children with trauma histories. Subsyndromal symptoms and childhood trauma showed poorer global functioning, estimated IQ, attention, and immediate verbal recall. Significant interactions exist between cognitive impairment and anxiety and depressive disorders and other psychiatric episodes

## Appendix C

**Table A3:** Breakdown of Themes.

First-Order Themes	Second-Order Themes	Third-Order Themes
Children in care have significant educational disadvantage	~ Chronicity of mental health is caused by trauma ~ subsyndromal symptoms of trauma contribute to cognitive impairment. Childhood mental health issues impact developmental domains and can complicate disabilities. Adult–child verbal interaction can support reductions in poor outcomes. Teachers of students with mental illness and developmental delay assessed as having a lower-quality teaching practice due to measuring quality against student test scores. Culturally sensitive workforce addressing the inequality related to minority groups and the over-representation of Indigenous students with trauma, disabilities, and mental illness.	Mental health conditions caused by trauma contribute to disability Disability is a risk factor for childhood abuse Inclusion and addressing the gap caused by integration Quality relational teaching practice needs to encompass social, emotional, and behavioural skills Culturally responsive, predictive, and child-centred innovations to address the inequity in Indigenous children being over-represented as vulnerable in education, health, and disabilities
School failure for children with trauma and disability is associated with long-term adverse consequences		
Mental health impacts caused by witnessing DV and neglect affect learning		
Significant barriers exist for teaching students with disabilities and additional behavioural needs caused by trauma		
One-third of children in care with trauma backgrounds qualified or received special education services. Disproportionately high rates of special education referrals for children who have experienced trauma		
Mental health implications for children in OOHC, further emphasising disabilities and developmental challenges		
Importance of multi-agency collaboration, effective screening processing of children with trauma histories, and identifying developmental concerns from an early age		
Significant developmental delay will occur as a result of complex trauma without intervention		
Domestic violence exposure and physical assaults impact on literacy skills and regulatory capacity in the classroom, furthering likelihood of mental health concerns		
Impacts on attachment have links to poor language development and communication issues		
Early exposure to abuse and neglect impacts on language acquisition developmental concerns		
Youth with disabilities who have experienced multiple adversities demonstrate mental health instability. Early childhood education can serve as a buffer to exacerbating disabilities and mental health instability		
Early intervention staff unskilled in how to promote the development of children with disabilities and who have developmental trauma		
Early childhood interventions are critical for children with vulnerabilities including disabilities, developmental trauma, and mental health complications		
High prevalence of mental health subsyndromal symptoms in children with disabilities		
Physical abuse, emotional abuse, and psychological injury impact on self-regulatory capacity		
Affect regulation has a significant role in developmental trauma		
Cumulative adversity equals clinical mental health levels		
Mental health symptoms coupled with disabilities are evident in classrooms. Concerns relating to if quality teaching for most equates to quality teaching for all		
Quality teaching needs to be inclusive of practices that accommodate all student cohorts including students with comorbid disabilities and mental illness		
Quality teaching practices needed to support inclusion of children with high-incidence disabilities such as ADHD, learning disabilities, and mental health complaints and not just integration		
Indigenous students require a relational pedagogical approach as a critical response to the complex lives in response to the underlying psychological dynamics including stress, grief, cultural estrangement, and developmental trauma		
